# The diagnostic pathway and time to diagnosis in ANCA-associated vasculitis: a retrospective study at a tertiary rheumatology center

**DOI:** 10.1007/s10067-026-08057-3

**Published:** 2026-03-31

**Authors:** Pauline Bussmann, Uta Kiltz, Hilal Kavruk, Philipp Sewerin, Johanna Mucke, Diana Vossen, David Kiefer, Ioana Andreica, Johanna Heuser, Judith Erkenberg, Panagiotis Ermeidis, Ralph Brinks, Xenofon Baraliakos, Anna Kernder

**Affiliations:** 1 Ruhr Universität Bochum, Rheumazentrum Ruhrgebiet, Rheumatology, Herne, Germany; 2 Medical Biometry and Epidemiology, Faculty of Health/School of Medicine, Herdecke University, Witten, Germany

**Keywords:** Anti-neutrophil cytoplasmic antibody-associated vasculitis, Delayed diagnosis, Referral and consultation

## Abstract

**Objective:**

To analyze the diagnostic pathway of patients with ANCA-associated vasculitis (AAV) and to identify factors associated with the time to diagnosis, including the role of referring and previously consulted medical specialties.

**Methods:**

This retrospective single-center study analyzed patients with granulomatosis with polyangiitis (GPA), microscopic polyangiitis (MPA), and eosinophilic granulomatosis with polyangiitis (EGPA) who received their diagnosis between 2014 and 2024. Data were extracted from medical records. Time to diagnosis was defined as the period between first AAV-related symptoms and confirmed diagnosis. Factors associated with diagnostic delay were analyzed using multivariate Cox regression. Temporal differences between subtypes were visualized using Kaplan–Meier curves.

**Results:**

A total of 216 patients were included (GPA *n* = 128; MPA *n* = 70; EGPA *n* = 18). Median time to diagnosis was numerically longest in EGPA (455 days [IQR 144–924]) compared with GPA (120 days [IQR 61334]) and MPA (153 days [IQR 90–366]). Renal involvement was statistically associated with a shorter time to diagnosis (HR 1.65, 95% CI 1.18–2.31, *p* = 0.004), whereas prior consultations with dermatologists (HR 0.41, *p* = 0.004), pulmonologists (HR 0.58, *p* = 0.003), and rheumatologists (HR 0.63, *p* = 0.003) showed longer delays. Higher BVAS and CRP levels statistically correlated with shorter diagnostic intervals.

**Conclusion:**

The diagnostic delay in AAV varies by disease subtype and clinical presentation. EGPA shows the numerically longest time to diagnosis, while renal involvement seems to facilitate earlier diagnosis. Enhanced awareness among non is essential to reduce diagnostic delay and prevent organ damage, although outcome measures were not assessed in our study.

**Table:** 

**Key Points** • *Diagnostic delay in ANCA-associated vasculitis varies between disease subtypes, organ involvement, and consulted specialties.* • *EGPA shows the numerically longest diagnostic delay, whereas renal involvement seems to facilitate earlier diagnosis.* • *Earlier recognition of AAV across specialties may reduce diagnostic delay and prevent organ damage.*

**Supplementary Information:**

The online version contains supplementary material available at 10.1007/s10067-026-08057-3.

## Introduction

ANCA-associated vasculitides (AAV) are systemic autoimmune diseases characterized by inflammation and necrosis of small blood vessels, typically affecting capillaries, venules, and arterioles [1, 2]. According to the 2012 revised Chapel Hill Consensus Conference nomenclature, AAV comprise three subtypes: granulomatosis with polyangiitis (GPA), microscopic polyangiitis (MPA), and eosinophilic granulomatosis with polyangiitis (EGPA) [1]. The reported incidence of AAV is approximately 3.3 per 100,000 persons per year, although marked geographical and methodological variations exist [3].

AAV are clinically heterogeneous and can affect any organ system. The upper and lower respiratory tract and kidneys are most commonly involved, but manifestations can also occur in the nervous system, skin, and other organs [2]. This wide clinical spectrum contributes to diagnostic complexity, often leading to delays in diagnosis. Prolonged time to diagnosis is clinically relevant, as it increases the risk of irreversible organ damage and mortality [4, 5]. Conversely, early diagnosis at lower disease activity levels enables less intensive immunosuppressive therapy, potentially reducing treatment-related adverse effects such as infections or cardiovascular complications.

AAV can cause irreversible organ damage early, especially when the kidneys or lungs are affected. G. Koo et al. has shown the damage measured using the Vasculitis Damage Index (VDI) is associated with long-term mortality [6]. Therefore, a delay in diagnosis is not only a measure of time but can represent a clinically significant window of opportunity in which preventable organ damage accumulates. However, the specific threshold at which a delay in diagnosis becomes clinically critical has not yet been clearly defined.

Previous studies have identified several factors contributing to diagnostic delay in vasculitis, including long referral times to specialized care, initial misdiagnosis, limited awareness of early manifestations, and non-specific disease presentations [4, 5]. Moreover, involvement of certain organ systems, such as the upper respiratory tract, has been associated with prolonged diagnostic intervals [5]. Although diagnostic algorithms have been proposed to facilitate early diagnosis [4], there remains substantial variability in clinical practice, particularly across healthcare systems.

To date, there are no data from Germany (one of the most populous countries in Europe) systematically evaluating the time to diagnosis, referring structures, and involved medical specialties in patients with AAV.

In Germany, healthcare is largely structured around a tiered system of outpatient and inpatient care. General practitioners act as the first point of contact for most patients and provide continuous primary care. They are responsible for initial assessment, management of common symptoms, and referral to outpatient specialists, such as pulmonologists, dermatologists, or otolaryngologists, when further evaluation is required. Outpatient specialists usually operate independently rather than within hospitals, which can lead to fragmented care when complex, multisystem symptoms occur. Hospital-based care is typically divided into primary, secondary, and tertiary levels, with tertiary centers providing specialized multidisciplinary services, including rheumatology and nephrology. Consequently, patients with atypical or multisystem disease often experience multiple sequential consultations before reaching tertiary care, potentially contributing to diagnostic delay.

Health care systems differ substantially regarding referral pathways and access to specialist care. Although diagnostic delay in AAV has been described in international cohorts, no study to date has systematically analyzed the role of referring institutions and the consultations of medical specialties within the German healthcare system.

Understanding these diagnostic pathways is essential to identify modifiable factors and improve early diagnosis. Therefore, the aim of this study was to analyze the duration from symptom onset to diagnosis and to characterize the referral patterns of patients newly diagnosed with AAV at a tertiary rheumatology center.

### Methods

#### Study design and patients

This retrospective study included patients with the AAV sub-entities GPA, MPA, and EGPA who were admitted as inpatients to our tertiary rheumatology center, covering a large catchment area. Eligible patients received their diagnosis of AAV at our center between 1 January 2014 and 30 December 2024. Confirmed diagnoses were ascertained by a team of rheumatology specialists according to the 2022 ACR/EULAR classification criteria.

Patients who had already been diagnosed with AAV before admission or before 1 January 2014, as well as those in whom vasculitis was excluded, were not included. Further exclusion criteria were incomplete or non-verifiable medical records.

#### Data collection and definitions

Retrospective data collection was carried out between 1 May 2024 and 30 March 2025. The duration of the diagnostic process was defined as the interval between the self-reported onset of first AAV-related symptoms and the date of confirmed diagnosis. When the exact day of symptom onset was uncertain, the 15th day of the reported month was used; if only the year was known, 30 June of that year was recorded (this concerns 12 (9.4%) of the patients with GPA, 9 (12.9%) of those with MPA and 4 (22.2%) of those with EGPA). This approach aimed to approximate the midpoint of the reported interval and minimize systematic over- or underestimation. Symptoms were considered initial manifestations when they presented with vasculitic or granulomatous features. In EGPA, isolated asthma or nasal polyps were not regarded as EGPA manifestations if they occurred without additional systemic or vasculitic features. For comparison purposes, the diagnostic delay that would result from including asthma and nasal polyps as initial manifestation was also determined.

Demographic variables (age at diagnosis, sex) and potential influencing factors such as number and type of previously consulted specialists and referring physicians were recorded. Both medical and surgical specialties (e.g., vascular surgery) were considered. Disease activity at diagnosis was assessed using the Birmingham Vasculitis Activity Score (BVAS V3.0). If BVAS was not documented, it was reconstructed from clinical records by MD students under the guidance of specialized rheumatologists. Only confirmed organ involvements were included.

Information on prior immunosuppressive or anti-inflammatory therapy (e.g., glucocorticoids, methotrexate, or other immunosuppressants) and previous rheumatological diagnoses (e.g., rheumatoid arthritis, fibromyalgia syndrome, or Raynaud’s phenomenon) was collected from clinical records. Laboratory results relevant for disease classification and activity assessment (including ANCA serology, eosinophil counts, CRP, ESR, renal function, and urinalysis) were extracted from the patients’ medical records.

In the case of previously administered immunosuppressive therapies, only the presence or absence of treatment was recorded; detailed information on dosage, duration, or indication was not consistently available due to the retrospective study design.

The variables were selected based on their clinical relevance, with age and gender considered as standard covariates and disease-specific factors (e.g., BVAS, organ involvement) taken into account to capture severity and diagnostic pathways.

The study was approved by the Ethics Committee of the Medical Association Westfalen-Lippe (approval number 2024–348-f-S).

#### Statistical analysis

Cox proportional hazards regression, adjusted for age and sex, was used to analyze factors influencing time to diagnosis. Variables included previously consulted and referring specialties, disease activity (BVAS, CRP), and organ involvement. The calendar year was included as a covariate in Cox regression to account for possible temporal trends during the study period. Data distribution was assessed visually. Normally distributed variables are reported as mean ± standard deviation (SD); non-normally distributed data are presented as median and interquartile range (IQR). Categorical variables are summarized as absolute numbers and percentages [*n* (%)]. Confidence bounds are reported on the 95% level (95% CI). A two-sided *p* < 0.05 was considered statistically significant.

To account for potential memory bias and data accuracy errors in retrospectively reported symptom onset, a Monte Carlo sensitivity analysis was performed. Gaussian noise corresponding to 2% of the observed variance in duration was added to the time-to-diagnosis variable over 500 iterations. The Cox proportional hazards model was recalculated in each iteration, and robustness was assessed by comparing the original hazard ratio with the mean simulated estimate and its 2.5th and 97.5th percentiles.

Temporal differences in time to diagnosis among AAV subtypes were visualized using Kaplan–Meier curves. Statistical analyses were performed with RStudio (version 2025.09.1 + 401) using the R packages survival, dplyr, ggplot2, survminer, scales, shiny, flextable, and officer.

## Results

### Patient characteristics

A total of 216 patients were included in the analysis, comprising 128 (59.3%) with GPA, 70 (32.4%) with MPA, and 18 (8.3%) with EGPA (Table 1). The mean age at diagnosis was 56.0 ± 16.0 years in GPA, 64.0 ± 13.1 years in MPA, and 60.0 ± 16.0 years in EGPA. Female patients constituted 51.0% (*n* = 65), 67.0% (*n* = 47), and 78.0% (*n* = 14) of the respective subgroups. Prior immunosuppressive therapy before the confirmed AAV diagnosis was documented in 53.1% (*n* = 68) of GPA, 45.7% (*n* = 32) of MPA, and 66.7% (*n* = 12) of EGPA cases, most commonly glucocorticoids.

**Table 1:** Demographic and clinical characteristics of patients with GPA, MPA, and EGPA

Characteristic	GPA (*n*=128)	MPA (*n*=70)	EGPA (*n*=18)
Age, mean ± SD (years)	56.0 ± 16.0	64.0 ± 13.1	60.0 ± 16.0
Female, n (%)	65 (51.0)	47 (67.0)	14 (78.0)
Prior immunosuppressive therapy, *n* (%)	68 (53.1)	32 (45.7)	12 (66.7)
BVAS, mean ± SD	9.0 ± 5.9	7.0 ± 4.9	8.0 ± 4.6
CRP, mean ± SD (mg/dl)	6.1 ± 11.6	4.7 ± 5.0	4.5 ± 6.1
Time to diagnosis, median [IQR] (days)	120.0 [61.0–334.0]	153.0 [90.0–366.0]	454.5 [144.0–924.0]

*BVAS* Birmingham Vasculitis Activity Score, *CRP* C-reactive protein, *IQR* interquartile range, *SD* standard deviation

The mean Birmingham Vasculitis Activity Score (BVAS) at diagnosis was 9.0 ± 5.9 in GPA, 7.0 ± 4.9 in MPA, and 8.0 ± 4.6 in EGPA. CRP levels were 6.1 ± 11.6 mg/dl, 4.7 ± 5.0 mg/dl, and 4.5 ± 6.1 mg/dl, respectively.

### Time to diagnosis

**Fig. 1:**
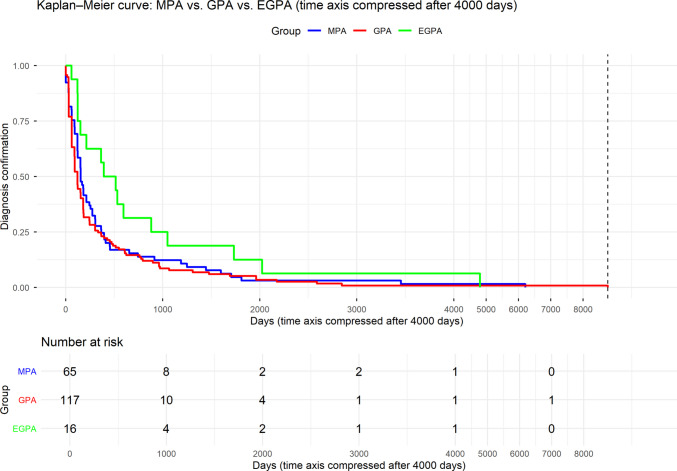
Kaplan–Meier curves illustrating the time to diagnosis in patients with GPA, MPA, and EGPA. The analysis demonstrates the longest diagnostic delay in patients with eosinophilic granulomatosis with polyangiitis (EGPA) compared with those with granulomatosis with polyangiitis (GPA) (*p* < 0.05) and microscopic polyangiitis (MPA) (*p* < 0.05).

The median time between first symptoms and confirmed diagnosis differed significantly across subtypes, with the numerically longest delay observed in EGPA (454.5 days [IQR 144.0–924.0]) compared with GPA (120.0 days [IQR 61.0–334.0]) and MPA (153.0 days [IQR 90.0–366.0]); however, the small subgroup in EGPA limits precision. The Kaplan–Meier analysis (Fig. 1) confirmed the significantly prolonged diagnostic delay in EGPA patients compared with GPA and MPA (*p* < 0.05).

In EGPA, an even longer diagnostic delay was observed when asthma and nasal polyps were considered an initial manifestation (625.0 days [IQR 327.0–1777.0]).

### Referring specialties and prior consultations

The most frequent referring specialties at the time of diagnosis were general practitioners, internists, and rheumatologists for GPA and MPA, whereas EGPA patients were more commonly referred by pulmonologists and cardiologists.

Before diagnosis, patients had typically consulted different specialists (mean 2.66, SD 1.3), with those with EGPA consulting the most specialists (mean 3.44, SD 1.72) (Tables 2 and S11). The most frequently visited specialties prior to diagnosis included general practitioners (51% (*n* = 111) of all cases), internists (52%, *n* = 112), rheumatologists (38%, *n* = 82), and otorhinolaryngologists (36%, *n* = 78). EGPA patients more often had contact with pulmonologists (50%, *n* = 9) and cardiologists (44%, *n* = 8).

**Table 2:** Most frequent referring and previously consulted specialties prior to diagnosis

Specialty	Frequency *n* (%)
General practice	111 (51.0)
Internal medicine	112 (52.0)
Rheumatology	82 (38.0)
Otorhinolaryngology (ENT)	78 (36.0)
Pulmonology	38 (18.0)
Cardiology	25 (12.0)

*ENT* Ear, Nose, Throat

### Predictors of diagnostic delay

Multivariate Cox regression identified several independent predictors of diagnostic delay (Table 3). Renal involvement at presentation was associated with a significantly shorter time to diagnosis (HR 1.65, 95% CI 1.18–2.31, *p* = 0.0036). Conversely, prior consultations with dermatologists (HR 0.41, 95% CI 0.22–0.76, *p* = 0.004) and pulmonologists (HR 0.58, 95% CI 0.41–0.83, *p* = 0.003) were associated with longer diagnostic delay.

**Table 3:** Multivariate Cox regression for predictors of diagnostic delay

Variable	HR (95% CI)	*p*-value
Renal involvement	1.65 (1.18–2.31)	0.0036
Pulmonary involvement	0.93 (0.69–1.23)	0.60
ENT involvement	0.92 (0.68–1.25)	0.59
Neurological involvement	1.13 (0.72–1.77)	0.61
Cutaneous involvement	0.80 (0.53–1.19)	0.27
Ocular involvement	1.35 (0.69–2.65)	0.38
Dermatology consultation	0.41 (0.22–0.76)	0.004
Pulmonology consultation	0.58 (0.41–0.83)	0.003
Rheumatology consultation	0.63 (0.47–0.86)	0.003
Referral by internist	1.44 (1.05–1.95)	0.022
BVAS (per unit increase)	1.06 (1.03–1.09)	<0.001
CRP (per mg/dl increase)	1.04 (1.02–1.05)	<0.001

*BVAS* Birmingham Vasculitis Activity Score, *CRP* C-reactive protein, *HR* hazard ratio, *CI* confidence interval, *ENT* Ear, Nose, Throat

Patients who had previously been evaluated by rheumatologists also experienced a significantly longer time to diagnosis (HR 0.63, 95% CI 0.47–0.86, *p* = 0.003). In contrast, referral by internists (HR 1.44, 95% CI 1.05–1.95, *p* = 0.022) was associated with a shorter diagnostic interval.

Higher BVAS scores (HR 1.06, 95% CI 1.03–1.09, *p* < 0.001) and elevated CRP levels (HR 1.04, 95% CI 1.02–1.05, *p* < 0.001) correlated with a more rapid diagnosis, indicating that higher disease activity facilitated an earlier diagnosis.

**Fig. 2:**
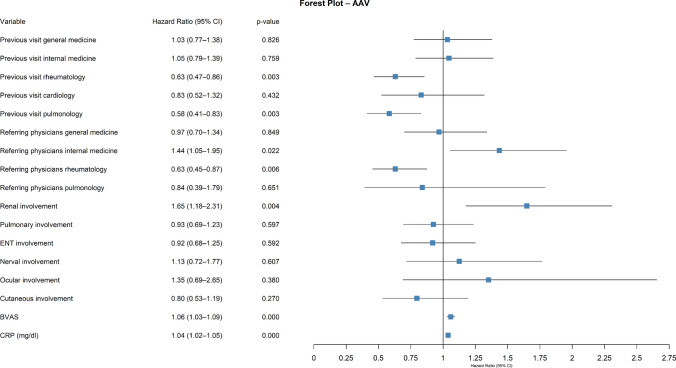
Forest plot of adjusted hazard ratios for predictors of diagnostic delay. The multivariate Cox regression analysis identified renal involvement as a predictor of a shorter time to diagnosis, whereas prior consultations with dermatologists or pulmonologists were associated with longer delays. Each point estimate represents the hazard ratio with corresponding 95% confidence intervals adjusted for age and sex

A forest plot summarizing the adjusted hazard ratios is presented in Fig. 2.

Adjustment for the calendar year did not reveal any significant temporal trend in the primary outcome, and inclusion of the calendar year did not significantly alter the estimated associations of the main variables (Tables S14a-S14c).

Overall, the effect estimates remained very stable under simulated temporal misclassifications, with most variables (85%) showing less than 5% deviation between the original and simulated hazard ratio estimates (Tables S13a-S13c).

## Discussion

In this retrospective study, we analyzed the diagnostic pathway of patients with AAV and identified factors associated with the time to diagnosis. Diagnostic delay varied substantially among AAV subtypes, with the numerically longest duration observed in EGPA.

Patients with renal involvement were diagnosed significantly earlier, most likely because renal manifestations such as acute kidney injury or abnormal urinalysis typically prompt investigation for systemic inflammation [2, 7]. In contrast, symptoms related to other organ systems, e.g., ENT manifestations, often evolve more gradually and may be interpreted as isolated or benign conditions, leading to delayed referral. Prior consultations with specialists, including dermatologists, pulmonologists, and rheumatologists, were statistically associated with longer diagnostic intervals. This finding suggests that patients with atypical or multisystem presentations are referred from outpatient rheumatology, dermatology, or pulmonology to tertiary centers frequently occurred before the definitive diagnostic work-up for AAV was completed. Together, these findings underline the persisting challenge of recognizing AAV in its early stages, despite advances in classification criteria and diagnostic tools [1–3].

Longer diagnostic delays observed in outpatient rheumatology, dermatology, or pulmonology consultations are more likely to reflect the complexity of the disease and atypical or organ-dominant manifestations than inefficiencies in these specialties. The involvement of specialists should be seen as an indicator of clinical complexity rather than a causal factor for delays. Reverse causality is also possible, as long-lasting or evolving symptoms may increase the likelihood of sequential referral to multiple specialists. These associations therefore highlight phenotypic patterns associated with more complex diagnostic trajectories.

Direct comparison of diagnostic delay between studies is difficult, as definitions of “time to diagnosis” and inclusion criteria vary considerably [5, 8]. A study including multiple vasculitis entities (66% AAV) reported a median delay of 7 months among predominantly Caucasian participants [4]. In contrast, an English registry–based study (2013–2015) described a median delay of 2.6 months (IQR 1.2–6.1) for AAV [9], while a Finnish cohort from 1996 to 2000 reported a 4-month delay for GPA [10], consistent with the interval observed in our German cohort.

The numerically prolonged diagnostic interval in EGPA likely reflects the clinical difficulty in distinguishing eosinophilic airway disease—manifesting as asthma or rhinosinusitis with hypereosinophilia—from the onset of systemic vasculitis [2, 4, 11]. This gradual transition often results in misclassification as allergic or respiratory disease and contributes to delayed diagnosis [12–14]. To avoid overestimation in this study, asthma and nasal polyps were not considered an initial manifestation if they occurred without additional systemic or vasculitic features, as the isolated occurrence of asthma and nasal polyps does not allow for a diagnosis. Nevertheless, these symptoms should be taken seriously, as they may precede systemic disease by many years and lead to an even longer time to diagnosis for patients.

Conversely, renal involvement typically triggers early diagnostic evaluation, as even mild abnormalities in kidney function raise suspicion for systemic disease [2, 7]. These observations are consistent with findings from the European Vasculitis Study Group (EUVAS), which identified the pattern of organ involvement, rather than demographic factors, as the strongest determinant of diagnostic timing and outcome [15, 16].

Diagnostic delay has repeatedly been associated with increased morbidity and mortality in AAV [17–20], without direct assessment of outcome measures in our cohort. Sreih et al. identified referral delay, initial misdiagnosis, and limited disease awareness as major determinants of late diagnosis [4].

Similarly, Dirikgil et al. demonstrated that patients with localized disease, particularly those with ENT involvement, experienced prolonged hospitalization prior to diagnosis [5].

Our results are in line with these findings and emphasize that early recognition of AAV remains highly dependent on clinical suspicion and interdisciplinary communication. The statistically observed association between prior rheumatology consultation and prolonged diagnostic interval likely reflects the complexity of differentiating early AAV from other autoimmune or inflammatory disorders [21].

The benefits of early diagnosis are well established. Prompt initiation of immunosuppressive therapy mitigates irreversible organ damage and reduces treatment-related toxicity by avoiding unnecessarily prolonged high-dose exposure [22]. Conversely, diagnostic delay facilitates disease progression and contributes to higher risks of infection and cardiovascular morbidity, although these points were not assessed in our study. Educational initiatives and structured referral pathways—similar to those implemented in other chronic rheumatic diseases—could be adapted for vasculitis to improve awareness among general practitioners and non-specialist physicians [23, 24].

Several limitations must be acknowledged. The retrospective, single-center design limits generalizability to other healthcare settings. As our center represents tertiary rheumatology care, patients managed exclusively in primary care or never referred to specialized units were not included. In particular, patients with renal involvement may have already received their initial diagnosis from nephrologists and continue to be treated there. Consequently, the longest diagnostic delays in undiagnosed or misdiagnosed individuals remain unquantified, introducing potential selection bias inherent to retrospective datasets [4, 5]. In addition, by limiting our cohort to a single tertiary center, individuals with more complex disease courses, atypical symptoms, or cases requiring referral to a specialized center may be disproportionately highly represented, potentially leading to longer observed diagnostic intervals compared to an unselected population-based cohort. Although the center served as the primary referral facility for a large and clearly defined geographic region, referral bias cannot be completely ruled out. Therefore, our results should be interpreted as reflecting the diagnostic trajectories of patients who ultimately require tertiary rheumatology care, rather than population-based estimates.

Although the 2022 ACR/EULAR classification criteria were not published until 2022, they were applied to all patients from 2014 onwards in order to create a homogenous and comparable cohort. Patients who did not meet these criteria were excluded.

Furthermore, the primary outcome is based on retrospective self-reported symptom onset and is therefore susceptible to memory bias and non-differential misclassification. However, symptom onset in this cohort was generally associated with clinically significant manifestations, making significant memory errors less likely. In addition, the dates of symptom onset reported by patients were systematically checked against medical records, and the final classification of onset was made by an experienced specialist. This physician-confirmed definition significantly reduces the likelihood of relevant misclassification. Furthermore, Monte Carlo sensitivity analysis demonstrated high robustness of effect estimates under simulated temporal inaccuracies, suggesting that moderate inaccuracies in dating onset likely did not substantially influence the study’s conclusions.

Although the primary Cox models were adjusted for only age and sex, additional analyses that included ANCA status as a covariate showed that it was a partial confounder. Inclusion of ANCA status slightly weakened the hazard ratio estimates, but the direction and significance of the associations remained unchanged (Tables S12a-S12c). These results suggest that the observed associations are robust to potential confounding factors due to ANCA status, while standard epidemiological practice supports reporting the primary models adjusted for age and sex. Therefore, residual confounding factors cannot be ruled out.

In EGPA, we were only able to analyze a small subgroup (*n* = 18) resulting in large interquartile ranges and limited statistical power. Therefore, estimates and comparisons for this subgroup are imprecise and should be interpreted with caution. The subgroup analyses were exploratory and descriptive, intended to illustrate the variability in diagnostic trajectories rather than provide definitive comparative conclusions. Larger, preferably multicenter studies are needed to confirm these findings.

Furthermore, the BVAS at the time of diagnosis was reconstructed retrospectively based on medical records by an MD student. Although data collection was performed using a standardized template and unclear cases were reviewed by an experienced rheumatologist, formal interrater reliability was not assessed, so a certain degree of measurement inaccuracy cannot be ruled out.

Due to the retrospective nature of the study, detailed categories of misdiagnoses, referral intervals, and diagnostic milestones could not be systematically recorded. Accordingly, the pathway data should be interpreted as exploratory and hypothesis-generating rather than a detailed representation of diagnostic trajectories. Future prospective studies with standardized data collection would be necessary to characterize misdiagnosis, referral times, and intervening diagnostic events more comprehensively.

In addition, differences in healthcare organization and referral structures between countries restrict direct cross-national comparison. On the other hand, our center is a big regional referral center for patients with complex rheumatologic conditions. While our study specifically examines diagnostic delay in the German healthcare system, some of the factors identified—such as the role of consulted and referring specialties—may also be relevant in other healthcare systems with similar access to specialists and referral pathways. Nevertheless, differences in the organization of healthcare, insurance systems, and access to specialists should be taken into account when extrapolating these results internationally. Prospective multicenter studies in different healthcare systems involving both primary and secondary care are needed to assess the generalization of our findings and warranted to confirm these findings and to identify system-level barriers to timely diagnosis.

Despite these limitations, our study provides valuable insights into the diagnostic process of AAV within a tertiary rheumatology setting. Identifying clinical and structural factors associated with delayed diagnosis is essential for developing targeted educational, diagnostic, and organizational strategies aimed at facilitating earlier recognition and improving patient outcomes.

## Supplementary Information

Below is the link to the electronic supplementary material.

ESM 1 (DOCX 15.5 MB)

## Data Availability

All data relevant to this article have been included in the text and the supplements. Supplementary data is available at *Clinical Rheumatology*.
