# Supplementary material for: The diagnostic pathway and time to diagnosis in ANCA-associated vasculitis: a retrospective study at a tertiary rheumatology center
Source: Clin Rheumatol. 2026 Mar 31;45(6):3565–73. doi: 10.1007/s10067-026-08057-3 (PMC13249735; doi:10.1007/s10067-026-08057-3)
Supplement: Supplementary file 1 — (DOCX 15.5 MB) [file 10067_2026_8057_MOESM1_ESM.docx]

Supplementary material:

Supplementary Table S1: Baseline characteristics of the study population

|  |  | **GPA** | **MPA** | **EGPA** |
| --- | --- | --- | --- | --- |
| Percentage | n (%) | 128 (59.3) | 70 (32.4) | 18 (8.3) |
| Age (years) | Mean  Standard deviation | 56  16.0 | 64  13.1 | 60  16.0 |
| Sex | Femal sex, n (%) | 65 (51) | 47 (67) | 14 (78) |
| Rheumatological preliminary diagnosis | Rheumatological prior diagnosis, n (%)  Raynaud`s syndrome, n (%)  rheumatoid arthritis, n (%)  Fibromyalgia syndrome, n (%)  No rheumatological prior diagnosis, n (%) | 19 (14.8)  4 (3.1)  7 (5.5)  2 (1.6)  109 (85.2) | 13 (18.6)  10 (7.0)  10 (7.0)  3 (4.3)  57 (81.4) | 2 (11.1)  1 (5.6)  1 (5.6)  0 (0.0)  16 (88.9) |
| Prior immuno-suppressive therapy | Recived previous therapy, n (%)  Received Glucocorticoids, n (%)  Received MTX, n (%)  Other Immunosuppressants, n (%) | 68 (53.1)  65 (50.8)  4 (3.1)  5 (3.9) | 32 (45.7)  29 (41.4)  2 (2.9)  1 (1.4) | 12 (66.7)  11 (61.1)  0 (0.0)  2 (11.1) |
| BVAS V3.0 | Mean  Standard deviation | 9  5.9 | 7  4.9 | 8  4.6 |
| CRP (mg/dl) | Mean  Standard deviation | 6.1  11.6 | 4.7  5.0 | 4.5  6.1 |
| Time to diagnosis | Median (Tage)  IQR (Tage) | 120.0  61.0–334.0 | 153.0  90.0–366.0 | 454.5  144.0–924.0 |
| Most frequent referring physicians | General medicine, n (%)  Internal medicine, n (%)  Rheumatology, n (%)  Cardiology, n (%)  Pneumonology, n (%) | 36 (28.1)  33 (25.8)  32 (25.0)  1 (0.8)  1 (0.8) | 17 (24.3)  22 (31.4)  22 (31.4)  1 (1.4)  2 (2.9) | 2 (11.1)  8 (44.4)  2 (11.1)  3 (16.7)  2 (11.1) |
| Previously consulted specialties | General medicine, n (%)  Internal medicine, n (%)  Rheumatology, n (%)  Cardiology, n (%)  Pneumonology n (%)  ENT, n (%)  Ophthalmology, n (%)  Neurology, n (%)  Nephrology, n (%)  Gastroenterology, n (%)  Hematology, n (%)  Geriatrics, n (%)  Angiology, n (%)  Vascular surgery, n (%)  Gynecology, n (%)  Urology, n (%)  Orthopedics, n (%)  Dermatology, n (%)  Dentistry, n (%)  Other specialties, n (%) | 65 (50.8)  65 (50.8)  47 (36.7)  11 (8.6)  30 (23.4)  47 (36.7)  10 (7.8)  15 (11.7)  6 (4.7)  12 (9.4)  2 (1.6)  1 (0.8)  1 (0.8)  3 (2.3)  0 (0.0)  1 (0.8)  14 (10.9)  9 (7.0)  0 (0.0)  6 (4.7) | 34 (48.6)  39 (55.7)  23 (32.9)  6 (8.6)  15 (21.4)  7 (10.0)  5 (7.1)  11 (15.7)  5 (7.1)  9 (12.9)  1 (1.4)  0 (0.0)  0 (0.0)  4 (5.7)  2 (2.9)  1 (1.4)  4 (5.7)  0 (0.0)  0 (0.0)  2 (2.9) | 4 (22.2)  13 (72.2)  3 (16.7)  8 (44.4)  9 (50.0)  5 (27.8)  1 (5.6)  5 (27.8)  2 (11.1)  3 (16.7)  5 (27.8)  0 (0.0)  0 (0.0)  0 (0.0)  0 (0.0)  0 (0.0)  0 (0.0)  4 (22.2)  0 (0.0)  0 (0.0) |

Abbreviation: BVAS, Birmingham Vasculitis Activity Score; CRP, C-reactive protein; ENT, Ear Nose Throat.

Supplementary Table S2a: Cox-Regression for consulted specialists in AAV

| Variable | hazard ratio | CI lower limit | CI upper limit | P value |
| --- | --- | --- | --- | --- |
| Previous visit others | 0.54 | 0.23 | 1.25 | 0.150 |
| Previous visit orthopedics | 0.76 | 0.46 | 1.26 | 0.292 |
| Previous visit dermatology | 0.41 | 0.22 | 0.76 | 0.004 |
| Previous visit neurology | 0.73 | 0.48 | 1.12 | 0.148 |
| Previous visit nephrology | 1.96 | 1.09 | 3.55 | 0.026 |
| Previous visit urology | 9.86 | 2.33 | 41.75 | 0.002 |
| Previous visit ophthalmology | 0.95 | 0.56 | 1.61 | 0.843 |
| Previous visit pulmonology | 0.58 | 0.41 | 0.83 | 0.003 |
| Previous visit gastroenterology | 0.65 | 0.41 | 1.04 | 0.075 |
| Previous visit ENT | 0.87 | 0.63 | 1.20 | 0.402 |
| Previous visit internal medicine | 1.05 | 0.79 | 1.39 | 0.759 |
| Previous visit geriatrics | 6.59 | 0.89 | 48.90 | 0.065 |
| Previous visit rheumatology | 0.63 | 0.47 | 0.86 | 0.003 |
| Previous visit hematology | 0.66 | 0.29 | 1.50 | 0.315 |
| Previous visit cardiology | 0.83 | 0.52 | 1.32 | 0.432 |
| Previous visit angiology | 0.93 | 0.13 | 6.65 | 0.939 |
| Previous visit gynecology | 0.96 | 0.24 | 3.91 | 0.958 |
| Previous visit vascular surgery | 1.89 | 0.88 | 4.08 | 0.105 |
| Previous visit general medicine | 1.03 | 0.77 | 1.38 | 0.826 |

Abbreviation: ENT, Ear Nose Throat.

Supplementary Table S2b: Cox-Regression for referring specialists in AAV

| Variable | Hazard ratio | CI lower limit | CI upper limit | P value |
| --- | --- | --- | --- | --- |
| Referring physicians general medicine | 0.97 | 0.70 | 1.34 | 0.849 |
| Referring physicians internal medicine | 1.44 | 1.05 | 1.95 | 0.022 |
| Referring physicians geriatrics | 4.88 | 0.64 | 37.30 | 0.127 |
| Referring physicians rheumatology | 0.63 | 0.45 | 0.87 | 0.006 |
| Referring physicians nephrology | 5.50 | 0.75 | 40.50 | 0.094 |
| Referring physicians gastroenterology | 0.93 | 0.13 | 6.75 | 0.947 |
| Referring physicians ENT | 1.43 | 0.69 | 2.96 | 0.336 |
| Referring physicians Pulmonology | 0.84 | 0.39 | 1.79 | 0.651 |
| Referring physicians neurology | 0.98 | 0.36 | 2.68 | 0.964 |
| Referring physicians ophthalmology | 1.15 | 0.16 | 8.33 | 0.888 |
| Referring physicians cardiology | 1.20 | 0.38 | 3.82 | 0.760 |
| Referring physicians orthopedics | 1.31 | 0.54 | 3.22 | 0.551 |
| Referring physicians vascular surgery | 1.36 | 0.19 | 9.80 | 0.759 |

Abbreviation: ENT, Ear Nose Throat.

Supplementary Table S3: Cox-Regression for organ involvement, BVAS, CRP in AAV

| Variable | Hazard ratio | CI lower limit | CI upper limit | p value |
| --- | --- | --- | --- | --- |
| Renal involvement | 1.65 | 1.18 | 2.31 | 0.004 |
| Pulmonary involvement | 0.93 | 0.69 | 1.23 | 0.597 |
| ENT involvement | 0.92 | 0.68 | 1.25 | 0.592 |
| Cardiac involvement | 1.01 | 0.62 | 1.65 | 0.958 |
| Cerebral involvement | 1.94 | 0.78 | 4.83 | 0.155 |
| Stroke | 1.28 | 0.18 | 9.22 | 0.808 |
| Vascular involvement | 0.83 | 0.47 | 1.46 | 0.512 |
| Nerval involvement | 1.13 | 0.72 | 1.77 | 0.607 |
| Muscular involvement | 0.95 | 0.55 | 1.65 | 0.865 |
| Tendon involvement | 2.39 | 0.33 | 17.28 | 0.387 |
| Gastrointestinal involvement | 1.45 | 0.53 | 3.96 | 0.468 |
| Ocular involvement | 1.35 | 0.69 | 2.65 | 0.380 |
| Cutaneous involvement | 0.80 | 0.53 | 1.19 | 0.270 |
| BVAS | 1.06 | 1.03 | 1.09 | <0.001 |
| CRP (mg/dl) | 1.04 | 1.02 | 1.05 | <0.001 |

Abbreviation: ENT, Ear Nose Throat; BVAS, Birmingham Vasculitis Activity Score; CRP, C-reactive protein.

Supplementary Table S4a: GPA consulted specialists

| Variable | Hazard ratio | CI lower limit | CI upper limit | p value |
| --- | --- | --- | --- | --- |
| Previous visit others | 0.55 | 0.20 | 1.56 | 0.26 |
| Previous visit orthopedics | 0.63 | 0.35 | 1.14 | 0.13 |
| Previous visit dermatology | 0.36 | 0.17 | 0.78 | 0.01 |
| Previous visit neurology | 0.50 | 0.25 | 0.98 | 0.04 |
| Previous visit nephrology | 2.31 | 0.91 | 5.89 | 0.08 |
| Previous visit urology | 4.07 | 0.54 | 30.94 | 0.17 |
| Previous visit ophthalmology | 1.04 | 0.52 | 2.07 | 0.90 |
| Previous visit pulmonology | 0.60 | 0.37 | 0.99 | 0.05 |
| Previous visit gastroenterology | 0.70 | 0.37 | 1.35 | 0.29 |
| Previous visit ENT | 0.98 | 0.67 | 1.44 | 0.93 |
| Previous visit internal medicine | 1.21 | 0.83 | 1.78 | 0.32 |
| Previous visit geriatrics | 4.98 | 0.65 | 38.07 | 0.12 |
| Previous visit rheumatology | 0.63 | 0.43 | 0.93 | 0.02 |
| Previous visit hematology | 0.62 | 0.08 | 4.48 | 0.63 |
| Previous visit cardiology | 0.90 | 0.45 | 1.82 | 0.77 |
| Previous visit angiology | 0.77 | 0.11 | 5.61 | 0.80 |
| Previous visit vascular surgery | 2.22 | 0.68 | 7.31 | 0.19 |
| Previous visit general medicine | 0.95 | 0.65 | 1.38 | 0.78 |

Supplementary Table S4a: GPA referring specialists

| Variable | Hazard ratio | CI lower limit | CI upper limit | p value |
| --- | --- | --- | --- | --- |
| Referring physicians general medicine | 0.88 | 0.58 | 1.33 | 0.55 |
| Referring physicians internal medicine | 1.47 | 0.97 | 2.24 | 0.07 |
| Referring physicians geriatrics | 4.88 | 0.64 | 37.30 | 0.13 |
| Referring physicians rheumatology | 0.67 | 0.43 | 1.03 | 0.07 |
| Referring physicians gastroenterology | 0.81 | 0.11 | 5.90 | 0.84 |
| Referring physicians ENT | 1.38 | 0.66 | 2.90 | 0.40 |
| Referring physicians pulmonology | 1.37 | 0.42 | 4.43 | 0.60 |
| Referring physicians neurology | 0.33 | 0.04 | 2.45 | 0.28 |
| Referring physicians cardiology | 5.21 | 0.69 | 39.37 | 0.11 |
| Referring physicians orthopedics | 1.98 | 0.60 | 6.48 | 0.26 |

Abbreviation: ENT, Ear Nose Throat.

Supplementary Table S5: GPA organ involvement, BVAS, CRP

| Variable | Hazard ratio | CI lower limit | CI upper limit | p-value |
| --- | --- | --- | --- | --- |
| Renal involvement | 1.61 | 1.02 | 2.53 | 0.04 |
| Pulmonary involvement | 1.05 | 0.72 | 1.54 | 0.80 |
| ENT involvement | 1.02 | 0.68 | 1.54 | 0.90 |
| Cardiac involvement | 1.10 | 0.49 | 2.47 | 0.82 |
| Cerebral involvement | 4.25 | 1.50 | 12.07 | 0.01 |
| Vascular involvement | 1.04 | 0.48 | 2.24 | 0.93 |
| Nerval involvement | 1.28 | 0.67 | 2.45 | 0.45 |
| Muscular involvement | 0.79 | 0.32 | 1.95 | 0.61 |
| Tendon involvement | 1.75 | 0.24 | 12.77 | 0.58 |
| Gastrointestinal involvement | 1.09 | 0.34 | 3.47 | 0.89 |
| Ocular involvement | 1.14 | 0.50 | 2.60 | 0.76 |
| Cutaneous involvement | 0.84 | 0.50 | 1.41 | 0.51 |
| BVAS | 1.05 | 1.02 | 1.09 | 0.003 |
| CRP (mg/dl) | 1.05 | 1.03 | 1.07 | <0.001 |

Abbreviation: BVAS, Birmingham Vasculitis Activity Score; CRP, C-reactive protein; ENT, Ear Nose Throat.

Supplementary Table S6a: MPA consulted specialists

| Variable | Hazard ratio | CI lower limit | CI upper limit | p value |
| --- | --- | --- | --- | --- |
| Previous visit others | 0.51 | 0.12 | 2.19 | 0.37 |
| Previous visit orthopedics | 1.11 | 0.40 | 3.09 | 0.85 |
| Previous visit neurology | 0.80 | 0.41 | 1.56 | 0.51 |
| Previous visit nephrology | 1.86 | 0.73 | 4.75 | 0.19 |
| Previous visit urology | 31.32 | 3.20 | 306.01 | 0.003 |
| Previous visit ophthalmology | 1.05 | 0.38 | 2.84 | 0.93 |
| Previous visit pulmonology | 0.55 | 0.29 | 1.05 | 0.07 |
| Previous visit gastroenterology | 0.68 | 0.31 | 1.47 | 0.33 |
| Previous visit ENT | 0.79 | 0.35 | 1.79 | 0.57 |
| Previous visit internal medicine | 0.90 | 0.54 | 1.47 | 0.66 |
| Previous visit rheumatology | 0.59 | 0.34 | 1.03 | 0.06 |
| Previous visit hematology | 0.57 | 0.08 | 4.17 | 0.58 |
| Previous visit cardiology | 1.27 | 0.53 | 3.08 | 0.59 |
| Previous visit gynecology | 0.92 | 0.22 | 3.82 | 0.91 |
| Previous visit vascular surgery | 1.50 | 0.54 | 4.20 | 0.44 |
| Previous visit general medicine | 1.15 | 0.67 | 1.98 | 0.61 |

Abbreviation: ENT, Ear Nose Throat.

Supplementary Table S6b: MPA referring specialists

| Variable | Hazard ratio | CI lower limit | CI upper limit | p value |
| --- | --- | --- | --- | --- |
| Referring physicians general medicine | 1.18 | 0.65 | 2.13 | 0.59 |
| Referring physicians internal medicine | 1.63 | 0.93 | 2.86 | 0.09 |
| Referring physicians rheumatology | 0.46 | 0.26 | 0.84 | 0.01 |
| Referring physicians nephrology | 6.02 | 0.75 | 48.52 | 0.09 |
| Referring physicians pulmonology | 0.91 | 0.21 | 3.94 | 0.90 |
| Referring physicians neurology | 1.96 | 0.45 | 8.61 | 0.37 |
| Referring physicians ophthalmology | 1.40 | 0.19 | 10.59 | 0.74 |
| Referring physicians cardiology | 1.45 | 0.19 | 11.16 | 0.72 |
| Referring physicians orthopedics | 0.96 | 0.23 | 3.99 | 0.95 |
| Referring physicians vascular surgery | 1.38 | 0.19 | 10.24 | 0.75 |

Supplementary Table S7: MPA organ involvement, BVAS, CRP

| Variable | Hazard ratio | CI lower limit | CI upper limit | p value |
| --- | --- | --- | --- | --- |
| Renal involvement | 1.40 | 0.80 | 2.47 | 0.24 |
| Pulmonary involvement | 1.04 | 0.62 | 1.75 | 0.87 |
| ENT involvement | 0.65 | 0.28 | 1.48 | 0.30 |
| Cardiac involvement | 1.20 | 0.51 | 2.86 | 0.67 |
| Stroke | 1.66 | 0.22 | 12.61 | 0.62 |
| Vascular involvement | 0.67 | 0.15 | 2.94 | 0.60 |
| Nerval involvement | 0.89 | 0.40 | 1.95 | 0.76 |
| Muscular involvement | 1.31 | 0.59 | 2.92 | 0.50 |
| Gastrointestinal involvement | 3.35 | 0.43 | 26.10 | 0.25 |
| Ocular involvement | 1.94 | 0.58 | 6.54 | 0.28 |
| Cutaneous involvement | 0.87 | 0.33 | 2.31 | 0.78 |
| BVAS | 1.05 | 0.99 | 1.10 | 0.11 |
| CRP (mg/dl) | 1.02 | 0.96 | 1.08 | 0.59 |

Abbreviation: BVAS, Birmingham Vasculitis Activity Score; CRP, C-reactive protein; ENT, Ear Nose Throat.

Supplementary Table S8a: EGPA consulted specialists

| Variable | Hazard ratio | CI lower limit | CI upper limit | p value |
| --- | --- | --- | --- | --- |
| Previous visit dermatology | 1.30 | 0.34 | 4.98 | 0.70 |
| Previous visit neurology | 2.84 | 0.75 | 10.70 | 0.12 |
| Previous visit nephrology | 1.59 | 0.24 | 10.57 | 0.63 |
| Previous visit ophthalmology | 0.47 | 0.06 | 3.82 | 0.48 |
| Previous visit pulmonology | 0.70 | 0.21 | 2.33 | 0.56 |
| Previous visit gastroenterology | 0.74 | 0.15 | 3.72 | 0.72 |
| Previous visit ENT | 0.24 | 0.05 | 1.21 | 0.08 |
| Previous visit internal medicine | 2.89 | 0.80 | 10.37 | 0.10 |
| Previous visit rheumatology | 0.16 | 0.03 | 0.89 | 0.04 |
| Previous visit hematology | 2.47 | 0.66 | 9.28 | 0.18 |
| Previous visit cardiology | 2.02 | 0.55 | 7.41 | 0.29 |
| Previous visit general medicine | 0.29 | 0.08 | 1.14 | 0.08 |

Abbreviation: ENT, Ear Nose Throat.

Supplementary Table S8b: EGPA referring specialists

| Variable | Hazard ratio | CI lower limit | CI upper limit | p value |
| --- | --- | --- | --- | --- |
| Referring physicians general medicine | 0.12 | 0.01 | 1.05 | 0.06 |
| Referring physicians internal medicine | 2.98 | 0.92 | 9.67 | 0.07 |
| Referring physicians rheumatology | 0.69 | 0.14 | 3.35 | 0.65 |
| Referring physicians pulmonology | 0.87 | 0.18 | 4.29 | 0.86 |
| Referring physicians neurology | 1.66 | 0.17 | 16.15 | 0.66 |
| Referring physicians cardiology | 6.45 | 0.40 | 103.15 | 0.19 |

Supplementary Table S9: EGPA organ involvement, BVAS, CRP

| Variable | Hazard ratio | CI lower limit | CI upper limit | p value |
| --- | --- | --- | --- | --- |
| Renal involvement | 2.68 | 0.44 | 16.16 | 0.28 |
| Pulmonary involvement | 0.19 | 0.04 | 0.95 | 0.04 |
| ENT involvement | 0.08 | 0.01 | 0.47 | 0.004 |
| Cardiac involvement | 1.00 | 0.30 | 3.32 | 1.00 |
| Cerebral involvement | 0.64 | 0.06 | 6.39 | 0.70 |
| Vascular involvement | 1.14 | 0.34 | 3.85 | 0.83 |
| Nerval involvement | 1.27 | 0.35 | 4.58 | 0.72 |
| Muscular involvement | 1.45 | 0.19 | 10.89 | 0.72 |
| Cutaneous involvement | 1.44 | 0.46 | 4.45 | 0.53 |
| BVAS | 1.11 | 0.96 | 1.28 | 0.15 |
| CRP (mg/dl) | 1.02 | 0.95 | 1.10 | 0.53 |

Abbreviation: BVAS, Birmingham Vasculitis Activity Score; CRP, C-reactive protein; ENT, Ear Nose Throat.

Supplementary Table S10: Number of previously consulted specialties prior to diagnosis

| Entity | Mean (standard deviation) |
| --- | --- |
| AAV | 2.66 (1.31) |
| GPA | 2.70 (1.33) |
| MPA | 2.40 (1.04) |
| EGPA | 3.44 (1.72) |

Abbreviation: AAV, ANCA-associated vasculitis; GPA, granulomatosis with polyangiitis; MPA, microscopic polyangiitis; EGPA, eosinophilic granulomatosis with polyangiitis

Supplementary Figure S11:


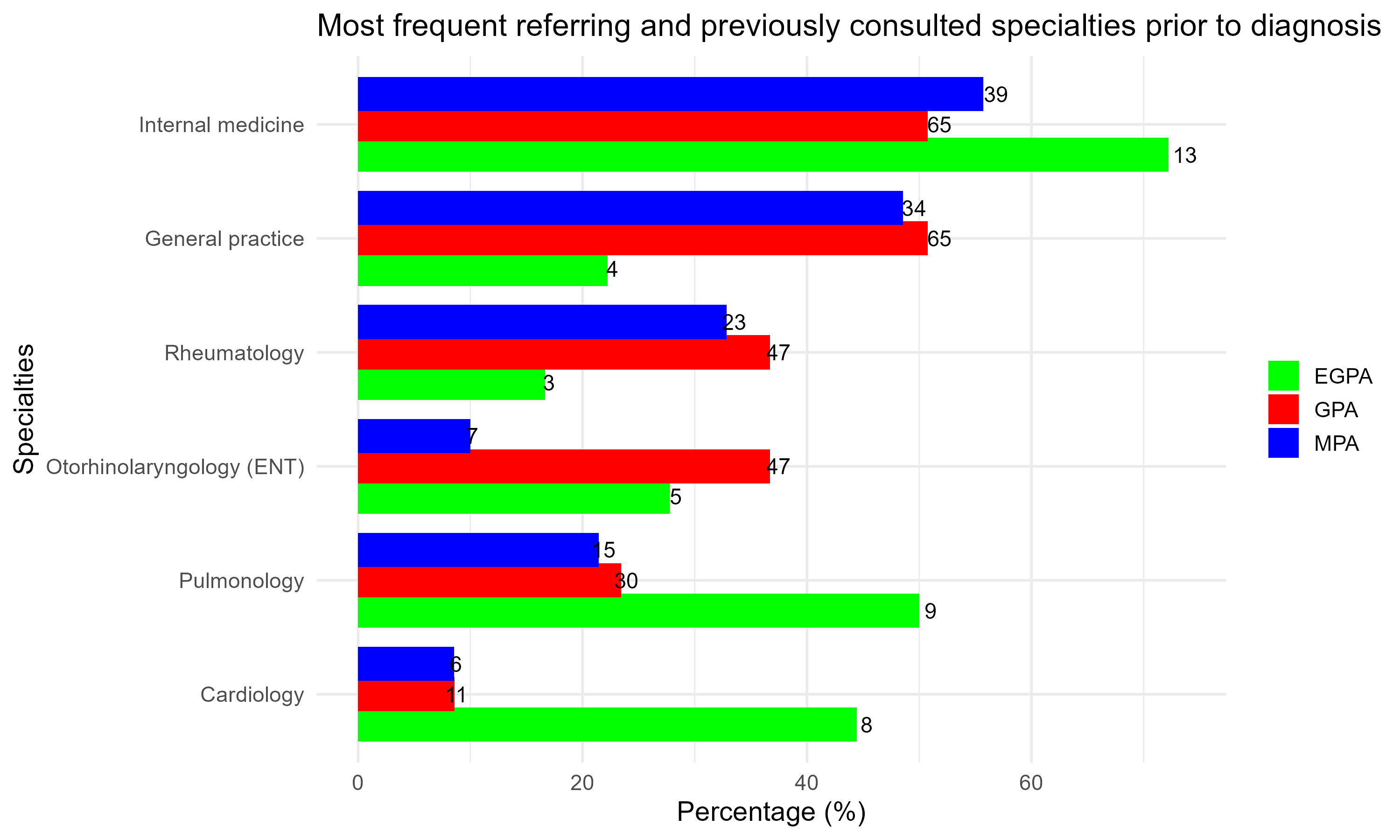


**Bar chart illustrating the most frequent referring and previously consulted specialties prior to diagnosis.**

The analysis identified internal medicine and general practice as the most referring and consulted specialties in all ANCA-associated vasculitis (AAV) sub-entities: granulomatosis with polyangiitis (GPA), microscopic polyangiitis (MPA) and eosinophilic granulomatosis with polyangiitis (EGPA).

Supplementary Table S12a: Cox-Regression with and without ANCA status for previously consulted specialties in AAV

| Previously consulted | HR basic | CI lower basic | CI upper basic | p-value basic | HR ANCA | CI lower ANCA | CI upper ANCA | p-value ANCA |
| --- | --- | --- | --- | --- | --- | --- | --- | --- |
| Others | 0.54 | 0.23 | 1.25 | 0.149000000 | 0.49 | 0.21 | 1.15 | 0.10100000 |
| Orthopedics | 0.76 | 0.46 | 1.26 | 0.290000000 | 0.82 | 0.49 | 1.36 | 0.44500000 |
| Dermatology | 0.41 | 0.22 | 0.76 | 0.004390000 | 0.37 | 0.19 | 0.71 | 0.00277000 |
| Neurology | 0.73 | 0.48 | 1.12 | 0.148000000 | 0.76 | 0.49 | 1.19 | 0.23600000 |
| Nephrology | 1.96 | 1.09 | 3.55 | 0.025700000 | 2.15 | 1.07 | 4.29 | 0.03050000 |
| Urology | 9.86 | 2.33 | 41.75 | 0.001890000 | 8.78 | 2.06 | 37.46 | 0.00335000 |
| Ophthalmology | 0.95 | 0.56 | 1.61 | 0.843000000 | 1.01 | 0.58 | 1.75 | 0.98300000 |
| Pulmonology | 0.58 | 0.41 | 0.83 | 0.002530000 | 0.58 | 0.41 | 0.84 | 0.00357000 |
| Gastro-enterology | 0.65 | 0.41 | 1.04 | 0.075200000 | 0.59 | 0.35 | 0.97 | 0.03760000 |
| ENT | 0.87 | 0.63 | 1.20 | 0.402000000 | 0.79 | 0.56 | 1.11 | 0.17900000 |
| Internal medicine | 1.05 | 0.79 | 1.39 | 0.759000000 | 1.06 | 0.79 | 1.43 | 0.67700000 |
| Geriatrics | 6.59 | 0.89 | 48.90 | 0.065100000 | 5.21 | 0.69 | 39.12 | 0.10900000 |
| Rheumatology | 0.63 | 0.47 | 0.86 | 0.002980000 | 0.62 | 0.46 | 0.85 | 0.00297000 |
| Hematology | 0.66 | 0.29 | 1.50 | 0.315000000 | 0.70 | 0.30 | 1.64 | 0.41600000 |
| Cardiology | 0.83 | 0.52 | 1.32 | 0.432000000 | 0.89 | 0.55 | 1.45 | 0.65200000 |
| Angiology | 0.93 | 0.13 | 6.65 | 0.939000000 | 0.72 | 0.10 | 5.26 | 0.75000000 |
| Gynecology | 0.96 | 0.24 | 3.91 | 0.958000000 | 1.04 | 0.25 | 4.26 | 0.95800000 |
| Vascular surgery | 1.89 | 0.88 | 4.08 | 0.105000000 | 2.14 | 0.93 | 4.91 | 0.07340000 |
| General medicine | 1.03 | 0.77 | 1.38 | 0.826000000 | 0.98 | 0.73 | 1.33 | 0.90500000 |

Abbreviation: ANCA, anti-neutrophil cytoplasmatic antibody; AAV, ANCA-associated vasculitis; HR, hazard ratio; CI, confidence interval; ENT, Ear Nose Throat.

Supplementary Table S12b: Cox-Regression with and without ANCA status for referring specialties in AAV

| Referring by | HR basic | CI lower basic | CI upper basic | p-value basic | HR ANCA | CI lower ANCA | CI upper ANCA | p-value ANCA |
| --- | --- | --- | --- | --- | --- | --- | --- | --- |
| General medicine | 0.97 | 0.70 | 1.34 | 0.849000000 | 0.93 | 0.66 | 1.30 | 0.67000000 |
| Internal medicine | 1.44 | 1.05 | 1.95 | 0.021900000 | 1.56 | 1.12 | 2.17 | 0.00874000 |
| Geriatrics | 4.88 | 0.64 | 37.30 | 0.127000000 | 4.56 | 0.59 | 35.01 | 0.14400000 |
| Rheumatology | 0.63 | 0.45 | 0.87 | 0.005720000 | 0.59 | 0.42 | 0.84 | 0.00372000 |
| Nephrology | 5.50 | 0.75 | 40.50 | 0.094000000 | 6.90 | 0.92 | 51.68 | 0.06010000 |
| Gastro-enterology | 0.93 | 0.13 | 6.75 | 0.947000000 | 0.82 | 0.11 | 5.95 | 0.84500000 |
| ENT | 1.43 | 0.69 | 2.96 | 0.336000000 | 1.28 | 0.61 | 2.69 | 0.51700000 |
| Pulmonology | 0.84 | 0.39 | 1.79 | 0.651000000 | 0.93 | 0.43 | 2.00 | 0.85800000 |
| Neurology | 0.98 | 0.36 | 2.68 | 0.964000000 | 0.88 | 0.32 | 2.44 | 0.80800000 |
| Ophthalmology | 1.15 | 0.16 | 8.33 | 0.888000000 | 1.36 | 0.19 | 9.96 | 0.76200000 |
| Cardiology | 1.20 | 0.38 | 3.82 | 0.758000000 | 1.48 | 0.46 | 4.80 | 0.51200000 |
| Orthopedics | 1.31 | 0.54 | 3.22 | 0.551000000 | 1.48 | 0.59 | 3.69 | 0.40200000 |
| Vascular surgery | 1.36 | 0.19 | 9.80 | 0.759000000 |  |  |  |  |

Abbreviation: ANCA, anti-neutrophil cytoplasmatic antibody; AAV, ANCA-associated vasculitis; HR, hazard ratio; CI, confidence interval; ENT, Ear Nose Throat.

Supplementary Table S12c: Cox-Regression with and without ANCA status for organ involvement in AAV

| Variable | HR basic | CI lower basic | CI upper basic | p-value basic | HR ANCA | CI lower ANCA | CI upper ANCA | p-value ANCA |
| --- | --- | --- | --- | --- | --- | --- | --- | --- |
| Renal involvement | 1.65 | 1.18 | 2.31 | 0.004 | 1.51 | 1.06 | 2.14 | 0.021 |
| Pulmonary involvement | 0.93 | 0.69 | 1.23 | 0.597 | 0.92 | 0.68 | 1.23 | 0.566 |
| ENT involvement | 0.92 | 0.68 | 1.25 | 0.592 | 0.78 | 0.55 | 1.10 | 0.155 |
| Cardiac involvement | 1.01 | 0.62 | 1.65 | 0.958 | 1.13 | 0.68 | 1.87 | 0.629 |
| Cerebral involvement | 1.94 | 0.78 | 4.83 | 0.1558 | 2.36 | 0.93 | 6.00 | 0.072 |
| Stroke | 1.28 | 0.18 | 9.23 | 0.8088 | 1.75 | 0.23 | 13.04 | 0.587 |
| Vascular involvement | 0.83 | 0.47 | 1.46 | 0.5128 | 0.97 | 0.54 | 1.75 | 0.927 |
| Neurological involvement | 1.13 | 0.72 | 1.77 | 0.6078 | 1.05 | 0.66 | 1.67 | 0.833 |
| Muscular involvement | 0.95 | 0.55 | 1.65 | 0.865 | 1.21 | 0.67 | 2.16 | 0.530 |
| Tendinous involvement | 2.39 | 0.33 | 17.28 | 0.387 |  |  |  |  |
| Gastrointestinal involvement | 1.45 | 0.53 | 3.96 | 0.468 | 1.56 | 0.57 | 4.29 | 0.387 |
| Ocular involvement | 1.35 | 0.69 | 2.65 | 0.380 | 1.15 | 0.56 | 2.34 | 0.708 |
| Cutaneous involvement | 0.80 | 0.53 | 1.19 | 0.270 | 0.82 | 0.54 | 1.25 | 0.358 |
| BVAS | 1.06 | 1.03 | 1.09 | <0.001 | 1.05 | 1.02 | 1.08 | 0.002 |
| CRP (mg/dl) | 1.04 | 1.02 | 1.05 | <0.001 | 1.04 | 1.02 | 1.05 | <0.001 |

Abbreviation: ANCA, anti-neutrophil cytoplasmatic antibody; AAV, ANCA-associated vasculitis; HR, hazard ratio; CI, confidence interval; ENT, Ear Nose Throat.

Supplementary Table S13a: Monte Carlo Sensitivity Analysis (Gaussian Noise, Variance = 2% of Duration) for previously consulted specialties in AAV

| Previously consulted | HR (original) | HR (MC mean) | Relative deviation | Stability classification* | HR (2.5^th^ percentile) | HR (97.5^th^ percentile) | p-value (original) | p-value (MC mean) |
| --- | --- | --- | --- | --- | --- | --- | --- | --- |
| Others | 0.54 | 0.54 | 0.45 | Very robust | 0.53 | 0.55 | 0.149 | 0.152 |
| Orthopedics | 0.76 | 0.76 | 0.12 | Very robust | 0.76 | 0.77 | 0.290 | 0.293 |
| Dermatology | 0.41 | 0.41 | 0.07 | Very robust | 0.41 | 0.41 | 0.004 | 0.004 |
| Neurology | 0.73 | 0.73 | 0.02 | Very robust | 0.72 | 0.74 | 0.148 | 0.148 |
| Nephrology | 1.96 | 1.95 | 0.59 | Very robust | 1.89 | 2.02 | 0.026 | 0.027 |
| Urology | 9.86 | 11.22 | 13.83 | Moderately stable | 7.57 | 19.75 | 0.002 | 0.002 |
| Ophthalmology | 0.95 | 0.95 | 0.03 | Very robust | 0.93 | 0.96 | 0.843 | 0.843 |
| Pulmonology | 0.58 | 0.58 | 0.11 | Very robust | 0.58 | 0.59 | 0.003 | 0.003 |
| Gastroenterology | 0.65 | 0.66 | 0.12 | Very robust | 0.65 | 0.66 | 0.075 | 0.076 |
| ENT | 0.87 | 0.87 | 0.25 | Very robust | 0.86 | 0.88 | 0.402 | 0.394 |
| Internal medicine | 1.05 | 1.05 | 0.03 | Very robust | 1.04 | 1.05 | 0.759 | 0.761 |
| Geriatrics | 6.59 | 7.86 | 19.30 | Moderately stable | 4.94 | 15.49 | 0.0651 | 0.059 |
| Rheumatology | 0.63 | 0.63 | 0.05 | Very robust | 0.63 | 0.64 | 0.003 | 0.003 |
| Hematology | 0.66 | 0.65 | 0.06 | Very robust | 0.65 | 0.66 | 0.315 | 0.315 |
| Cardiology | 0.83 | 0.83 | 0.29 | Very robust | 0.82 | 0.85 | 0.432 | 0.439 |
| Angiology | 0.93 | 0.92 | 0.94 | Very robust | 0.88 | 0.95 | 0.939 | 0.932 |
| Gynecology | 0.96 | 0.96 | 0.73 | Very robust | 0.92 | 0.99 | 0.958 | 0.950 |
| Vascular surgery | 1.89 | 1.90 | 0.66 | Very robust | 1.82 | 2.00 | 0.105 | 0.102 |
| General medicine | 1.03 | 1.03 | 0.14 | Very robust | 1.02 | 1.04 | 0.826 | 0.833 |

Abbreviation: AAV, ANCA-associated vasculitis; HR, hazard ratio; MC mean, the Monte Carlo mean estimate; ENT, Ear, Nose, Throat.

*Robustness was classified based on the relative deviation between the original hazard ratio and the Monte Carlo mean estimate:
<5% = very robust, 5–10% = robust, 10–20% = moderately stable, >20% = unstable.

Supplementary Table S13b: Monte Carlo Sensitivity Analysis (Gaussian Noise, Variance = 2% of Duration) for referring specialties in AAV

| Referring by | HR (original) | HR (MC mean) | Relative deviation | Stability classification* | HR (2.5^th^ percentile) | HR (97.5^th^ percentile) | p-value (original) | p-value (MC mean) |
| --- | --- | --- | --- | --- | --- | --- | --- | --- |
| General medicine | 0.97 | 0.97 | 0.33 | Very robust | 0.96 | 0.98 | 0.849 | 0.833 |
| Internal medicine | 1.44 | 1.43 | 0.04 | Very robust | 1.42 | 1.45 | 0.022 | 0.022 |
| Geriatrics | 4.88 | 6.15 | 25.95 | Unstable | 3.47 | 14.34 | 0.127 | 0.119 |
| Rheumatology | 0.63 | 0.63 | 0.05 | Very robust | 0.63 | 0.64 | 0.006 | 0.006 |
| Nephrology | 5.50 | 6.47 | 17.51 | Moderately stable | 4.11 | 12.03 | 0.094 | 0.088 |
| Gastroenterology | 0.93 | 0.97 | 3.73 | Very robust | 0.93 | 1.07 | 0.947 | 0.964 |
| ENT | 1.43 | 1.43 | 0.26 | Very robust | 1.39 | 1.48 | 0.336 | 0.333 |
| Pulmonology | 0.84 | 0.84 | 0.44 | Very robust | 0.83 | 0.86 | 0.651 | 0.659 |
| Neurology | 0.98 | 0.98 | 0.06 | Very robust | 0.97 | 0.99 | 0.964 | 0.965 |
| Ophthalmology | 1.15 | 1.18 | 2.39 | Very robust | 1.10 | 1.31 | 0.888 | 0.870 |
| Cardiology | 1.20 | 1.20 | 0.27 | Very robust | 1.15 | 1.27 | 0.758 | 0.755 |
| Orthopedics | 1.31 | 1.30 | 0.73 | Very robust | 1.27 | 1.33 | 0.551 | 0.562 |
| Vascular surgery | 1.36 | 1.36 | 0.03 | Very robust | 1.36 | 1.37 | 0.759 | 0.759 |

Abbreviation: AAV, ANCA-associated vasculitis; HR, hazard ratio; MC mean, the Monte Carlo mean estimate; ENT, Ear, Nose, Throat.

*Robustness was classified based on the relative deviation between the original hazard ratio and the Monte Carlo mean estimate:
<5% = very robust, 5–10% = robust, 10–20% = moderately stable, >20% = unstable.

Supplementary Table S13c: Monte Carlo Sensitivity Analysis (Gaussian Noise, Variance = 2% of Duration) for organ involvement, BVAS and CRP in AAV

| Variable | HR (original) | HR (MC mean) | Relative deviation | Stability classification* | HR (2.5^th^ percentile) | HR (97.5^th^ percentile) | p-value (original) | p-value (MC mean) |
| --- | --- | --- | --- | --- | --- | --- | --- | --- |
| Renal involvement | 1.65 | 1.65 | 0.37 | Very robust | 1.63 | 1.68 | 0.004 | 0.003 |
| Pulmonary involvement | 0.93 | 0.92 | 0.06 | Very robust | 0.92 | 0.93 | 0.597 | 0.595 |
| ENT involvement | 0.92 | 0.91 | 0.49 | Very robust | 0.91 | 0.92 | 0.592 | 0.571 |
| Cardiac involvement | 1.01 | 1.01 | 0.30 | Very robust | 1.00 | 1.03 | 0.958 | 0.966 |
| Cerebral involvement | 1.94 | 1.92 | 1.02 | Very robust | 1.81 | 2.02 | 0.155 | 0.162 |
| Stroke | 1.28 | 1.25 | 2.18 | Very robust | 1.13 | 1.32 | 0.808 | 0.826 |
| Vascular involvement | 0.83 | 0.83 | 0.17 | Very robust | 0.82 | 0.84 | 0.512 | 0.516 |
| Neurological involvement | 1.13 | 1.12 | 0.36 | Very robust | 1.10 | 1.14 | 0.607 | 0.619 |
| Muscular involvement | 0.95 | 0.95 | 0.01 | Very robust | 0.94 | 0.97 | 0.865 | 0.865 |
| Tendinous involvement | 2.39 | 2.48 | 3.55 | Very robust | 2.21 | 2.91 | 0.387 | 0.372 |
| Gastrointestinal involvement | 1.45 | 1.42 | 2.35 | Very robust | 1.35 | 1.48 | 0.468 | 0.497 |
| Ocular involvement | 1.35 | 1.35 | 0.03 | Very robust | 1.31 | 1.40 | 0.380 | 0.382 |
| Cutaneous involvement | 0.80 | 0.80 | 0.14 | Very robust | 0.79 | 0.81 | 0.270 | 0.273 |
| BVAS | 1.06 | 1.06 | 0.01 | Very robust | 1.06 | 1.06 | <0.001 | <0.001 |
| CRP (mg/dl) | 1.04 | 1.04 | 0.01 | Very robust | 1.04 | 1.04 | <0.001 | <0.001 |

Abbreviation: AAV, ANCA-associated vasculitis; HR, hazard ratio; MC mean, the Monte Carlo mean estimate; ENT, Ear, Nose, Throat; BVAS, Birmingham Vasculitis Activity Score; CRP, C-reactive protein.

*Robustness was classified based on the relative deviation between the original hazard ratio and the Monte Carlo mean estimate:
<5% = very robust, 5–10% = robust, 10–20% = moderately stable, >20% = unstable.

Supplementary Table S14a: Cox regression with and without adjustment for calendar year for previously consulted specialties in AAV

| Previously consulted | HR | CI lower | CI upper | p-value | HR year | CI year lower | CI year upper | p-value year |
| --- | --- | --- | --- | --- | --- | --- | --- | --- |
| Others | 0.53 | 0.23 | 1.22 | 0.134 | 1.03 | 0.98 | 1.08 | 0.255 |
| Orthopedics | 0.76 | 0.46 | 1.25 | 0.273 | 1.03 | 0.98 | 1.08 | 0.277 |
| Dermatology | 0.42 | 0.22 | 0.77 | 0.006 | 1.02 | 0.97 | 1.07 | 0.509 |
| Neurology | 0.72 | 0.47 | 1.11 | 0.134 | 1.03 | 0.98 | 1.08 | 0.263 |
| Nephrology | 2.11 | 1.15 | 3.84 | 0.015 | 1.03 | 0.99 | 1.08 | 0.178 |
| Urology | 9.94 | 2.35 | 42.11 | 0.002 | 1.03 | 0.98 | 1.08 | 0.290 |
| Ophthalmology | 0.93 | 0.55 | 1.58 | 0.787 | 1.03 | 0.98 | 1.08 | 0.289 |
| Pulmonology | 0.59 | 0.42 | 0.84 | 0.003 | 1.02 | 0.97 | 1.07 | 0.486 |
| Gastro-enterology | 0.67 | 0.42 | 1.07 | 0.0939 | 1.02 | 0.97 | 1.07 | 0.414 |
| ENT | 0.87 | 0.63 | 1.19 | 0.38 | 1.03 | 0.98 | 1.08 | 0.282 |
| Internal medicine | 1.06 | 0.80 | 1.40 | 0.706 | 1.03 | 0.98 | 1.08 | 0.285 |
| Geriatrics | 7.25 | 0.97 | 54.20 | 0.054 | 1.03 | 0.98 | 1.08 | 0.263 |
| Rheumatology | 0.61 | 0.45 | 0.83 | 0.002 | 1.04 | 0.99 | 1.09 | 0.144 |
| Hematology | 0.71 | 0.30 | 1.65 | 0.423 | 1.02 | 0.97 | 1.07 | 0.420 |
| Cardiology | 0.82 | 0.51 | 1.30 | 0.388 | 1.03 | 0.98 | 1.08 | 0.268 |
| Angiology | 0.87 | 0.12 | 6.26 | 0.888 | 1.03 | 0.98 | 1.08 | 0.293 |
| Gynecology | 0.99 | 0.24 | 4.01 | 0.986 | 1.03 | 0.98 | 1.08 | 0.297 |
| Vascular surgery | 1.89 | 0.88 | 4.08 | 0.105 | 1.03 | 0.98 | 1.08 | 0.298 |
| General medicine | 1.06 | 0.79 | 1.42 | 0.704 | 1.03 | 0.98 | 1.08 | 0.275 |

Abbreviation: AAV, ANCA-associated vasculitis; HR, hazard ratio; CI, confidence interval; ENT, Ear, Nose, Throat.

Supplementary Table S14b: Cox regression with and without adjustment for calendar year for referring specialties in AAV

| Referring by | HR | CI lower | CI upper | p-value | HR year | CI year lower | CI year upper | p-value year |
| --- | --- | --- | --- | --- | --- | --- | --- | --- |
| General medicine | 0.97 | 0.71 | 1.35 | 0.875 | 1.02 | 0.98 | 1.08 | 0.323 |
| Internal medicine | 1.47 | 1.08 | 2.01 | 0.015 | 1.03 | 0.98 | 1.08 | 0.201 |
| Geriatrics | 5.51 | 0.71 | 42.98 | 0.103 | 1.03 | 0.96 | 1.10 | 0.397 |
| Rheumatology | 0.62 | 0.44 | 0.86 | 0.004 | 1.03 | 0.98 | 1.08 | 0.190 |
| Nephrology | 5.96 | 0.81 | 44.08 | 0.081 | 1.03 | 0.98 | 1.08 | 0.291 |
| Gastro-enterology | 0.97 | 0.13 | 7.02 | 0.977 | 1.02 | 0.98 | 1.08 | 0.321 |
| ENT | 1.35 | 0.64 | 2.83 | 0.425 | 1.02 | 0.97 | 1.07 | 0.391 |
| Pulmonology | 0.90 | 0.41 | 1.94 | 0.780 | 1.02 | 0.97 | 1.08 | 0.356 |
| Neurology | 0.96 | 0.35 | 2.63 | 0.933 | 1.02 | 0.98 | 1.08 | 0.319 |
| Ophthalmology | 1.04 | 0.14 | 7.60 | 0.967 | 1.02 | 0.98 | 1.08 | 0.324 |
| Cardiology | 1.14 | 0.36 | 3.64 | 0.828 | 1.02 | 0.98 | 1.08 | 0.331 |
| Orthopedics | 1.32 | 0.54 | 3.23 | 0.548 | 1.02 | 0.98 | 1.08 | 0.319 |
| Vascular medicine | 1.62 | 0.22 | 11.96 | 0.638 | 1.03 | 0.98 | 1.08 | 0.295 |

Abbreviation: AAV, ANCA-associated vasculitis; HR, hazard ratio; CI, confidence interval; ENT, Ear, Nose, Throat.

Supplementary Table S14c: Cox regression with and without adjustment for calendar year for organ involvement, BVAS and CRP in AAV

| Variable | HR | CI lower | CI upper | p-value | HR year | CI lower year | CI upper year | p-value year |
| --- | --- | --- | --- | --- | --- | --- | --- | --- |
| Renal involvement | 1.63 | 1.16 | 2.28 | 0.0047 | 1.02 | 0.97 | 1.07 | 0.414 |
| Pulmonary involvement | 0.90 | 0.67 | 1.21 | 0.484 | 1.03 | 0.98 | 1.08 | 0.253 |
| ENT involvement | 0.89 | 0.65 | 1.22 | 0.481 | 1.03 | 0.98 | 1.08 | 0.253 |
| Cardiac involvement | 1.04 | 0.64 | 1.70 | 0.867 | 1.03 | 0.98 | 1.08 | 0.290 |
| Cerebral involvement | 2.00 | 0.80 | 4.98 | 0.138 | 1.03 | 0.98 | 1.08 | 0.268 |
| Stroke | 1.15 | 0.16 | 8.39 | 0.890 | 1.03 | 0.98 | 1.08 | 0.303 |
| Vascular involvement | 0.82 | 0.47 | 1.44 | 0.494 | 1.03 | 0.98 | 1.08 | 0.287 |
| Neurological involvement | 1.10 | 0.70 | 1.73 | 0.684 | 1.03 | 0.98 | 1.08 | 0.317 |
| Muscular involvement | 0.97 | 0.56 | 1.69 | 0.923 | 1.03 | 0.98 | 1.08 | 0.300 |
| Tendinous involvement | 2.26 | 0.31 | 16.41 | 0.418 | 1.03 | 0.98 | 1.08 | 0.309 |
| Gastrointestinal involvement | 1.47 | 0.54 | 4.02 | 0.452 | 1.03 | 0.98 | 1.08 | 0.288 |
| Ocular involvement | 1.35 | 0.69 | 2.64 | 0.388 | 1.03 | 0.98 | 1.08 | 0.300 |
| Cutaneous involvement | 0.82 | 0.55 | 1.24 | 0.355 | 1.02 | 0.97 | 1.07 | 0.400 |
| BVAS | 1.06 | 1.03 | 1.09 | <0.001 | 1.02 | 0.97 | 1.07 | 0.425 |
| CRP (mg/dl) | 1.04 | 1.02 | 1.05 | <0.001 | 1.02 | 0.97 | 1.07 | 0.482 |

Abbreviation: Abbreviation: AAV, ANCA-associated vasculitis; HR, hazard ratio; CI, confidence interval; ENT, Ear, Nose, Throat.
